# Molecular prevalence and genetic diversity of *Bartonella* spp. in stray cats of İzmir, Turkey

**DOI:** 10.1186/s13071-022-05431-3

**Published:** 2022-08-29

**Authors:** Ahmet Efe Köseoğlu, Hüseyin Can, Mervenur Güvendi, Muhammet Karakavuk, Pumla Manyatsi, Sedef Erkunt Alak, Aysu Değirmenci Döşkaya, Aytül Gül, Mert Döşkaya, Adnan Yüksel Gürüz, Cemal Ün

**Affiliations:** 1 Department of Molecular Biology and Genetics, Faculty of Engineering and Natural Sciences, Biruni University, Istanbul, Turkey; 2 Department of Biology Molecular Biology Section, Faculty of Science, Ege University, Izmir, Turkey; 3 Ege University Ödemis Vocational School, Izmir, Turkey; 4 Department of Parasitology, Faculty of Medicine, Ege University, Izmir, Turkey; 5 Department of Bioengineering, Faculty of Engineering, Ege University, Izmir, Turkey

**Keywords:** *Bartonella henselae*, *Bartonella clarridgeiae*, *Bartonella koehlerae*, Cat, Prevalence, Haplotype/genetic diversity

## Abstract

**Background:**

*Bartonella* spp. are vector-borne pathogens that cause zoonotic infections in humans. One of the most well-known of these is cat-scratch disease caused by *Bartonella henselae* and *Bartonella clarridgeiae*, with cats being the major reservoir for these two bacteria. Izmir, Turkey is home to many stray cats, but their potential role as a reservoir for the transmission of *Bartonella* to humans has not been investigated yet. Therefore, the aim of this study was to investigate the prevalence of *Bartonella* species and their genetic diversity in stray cats living in Izmir.

**Methods:**

Molecular prevalence of *Bartonella* spp. in stray cats (*n* = 1012) was investigated using a PCR method targeting the *16S-23S* internal transcribed spacer gene (ITS), species identification was performed by sequencing and genetic diversity was evaluated by haplotype analysis.

**Results:**

Analysis of the DNA extracted from 1012 blood samples collected from stray cats revealed that 122 samples were *Bartonella-*positive, which is a molecular prevalence of 12.05% (122/1012; 95% confidence interval [CI] 10.1–14.2%). Among the *Bartonella-*positive specimens, 100 (100/122; 81.96%) were successfully sequenced, and *B. henselae* (45/100; 45%), *B. clarridgeiae* (29/100; 29%) and *Bartonella koehlerae* (26/100; 26%) were identified by BLAST and phylogenetic analyses. High genetic diversity was detected in *B. clarridgeiae* with 19 haplotypes, followed by *B. henselae* (14 haplotypes) and *B. koehlerae* (8 haplotypes).

**Conclusions:**

This comprehensive study analyzing a large number of samples collected from stray cats showed that *Bartonella* species are an important source of infection to humans living in Izmir. In addition, high genetic diversity was detected within each *Bartonella* species.

**Supplementary Information:**

The online version contains supplementary material available at 10.1186/s13071-022-05431-3.

## Background

*Bartonella* spp. are Gram-negative bacteria from the family *Bartonellaceae* with more than 23 defined species that infect domestic and wild mammals and humans [1–3]. *Bartonella henselae*, *B. clarridgeiae*, *B. quintana*, and *B. bacilliformis* are the most common species associated with human diseases [1, 2]. Among these, *B. henselae* and *B. clarridgeiae* cause cat-scratch disease while *B. quintana* causes trench fever disease. Both diseases are called bartonellosis and manifest with symptoms such as fever, bacteremia, bacillary angiomatosis and endocarditis [2, 4]. *Bartonella koehlerae, B. elizabethae* and *B. alsatica* also have been associated with sporadic cases of endocarditis in humans [5, 6].

The principal reservoir hosts for *B. henselae*, *B. clarridgeiae* and *B. koehlerae* are domestic cats [3, 7, 8]. Other *Bartonella* species, such as *B. rochalimae*, *B. elizabethae*, *B. quintana* and *B. grahamii*, have been detected in cats [9]. Cats can become infected with many *Bartonella* species, but they usually show no symptoms. However, uveitis and endocarditis have been associated with *B. henselae* infection in cats [10], and lymphadenopathy, fever and neurological signs have been reported in experimentally infected cats [11].

Various diagnostic methods are currently in use to diagnose bartonellosis and/or applied during epidemiological surveys, such as culture, PCR assay, histopathology and serology. Among these methods, PCR assays targeting *Bartonella*-specific gene sequences have become a very important tool for the diagnosis of *Bartonella* species, which are very difficult to isolate from blood or tissue samples [11, 12]. The *16S* ribosomal RNA (rRNA) gene was initially used during the molecular diagnosis of *Bartonella* species, but was subsequently shown that it could not provide sufficient distinction in phylogenetic analysis at the species level [13]. More reliable phylogenetic results and species distinctions are obtained by analyzing the 16S–23S rDNA intergenic spacer (ITS) and *gltA* genes [13–15].

The prevalence of *Bartonella* in cats has been reported to vary from 4% to 70% using blood culture methods [16], and the seroprevalence of antibodies against *Bartonella* in cats also varies, ranging from 0 to 80%. An increased prevalence has been especially reported in warmer regions; for example, in a study conducted in California, the seroprevalence in cats was 80% compared to 0% in a study conducted in Norway [17, 18]. In studies conducted in the Middle East, including Saudi Arabia and Iraq, seroprevalence rates in cats were found to be 15% for *B. henselae* and 12.6% for *B. clarridgeiae* whereas *Bartonella* DNA positivity was 9.25% [19, 20]. In Iran, *Bartonella* DNA positivity was reported to vary from 14% to 74.2% in dogs [21, 22]. In the same region, *Bartonella* DNA positivity was reported to be 7.14% and 1.42% in nail and saliva samples collected from cats [23]. In Turkey, the prevalence of *Bartonella* was found to be 9.4% in domestic cats by blood culture methods but seroprevalence reached up to 40% [7, 8].

Since the zoonotic transmission of *Bartonella* occurs by a cat scratch or through the bite of a vector, the prevalence of *Bartonella* in stray cats that are in close contact with humans is frequently being screened in many countries [8, 9, 24–26]. Although the weather is very hot in Izmir, Turkey, especially during the summer, and the city is home to many stray cats, the prevalence of *Bartonella* and species of *Bartonella* have not been investigated. Therefore, the aim of this study was to investigate the molecular prevalence of *Bartonella* in a large number of blood samples collected from stray cats and to sequence the positive samples for species identification. In addition, genetic diversity within each detected species was investigated by haplotype analysis.

## Methods

### Blood samples

Blood samples (*n* = 1012) were collected from stray cats in Izmir city that had been brought to veterinary clinics located in the districts of Balçova (*n* = 110), Bayraklı (*n* = 43), Bornova (*n* = 48), Buca (*n* = 54), Çiğli (*n* = 6), Gaziemir (*n* = 4), Güzelbahçe (*n* = 10), Karabağlar (*n* = 115), Karşıyaka (*n* = 4), Konak (*n* = 614) and Narlıdere (*n* = 4). These stray cats were captured by persons in an animal friendly manner, without any harm being inflicted on the animal, and brought to the clinics for sterilization. The probability sampling method was used for sampling.

### Conventional PCR

DNA was isolated from the blood samples collected from the stray cats using a commercial kit (Qiagen DNA Extraction Kit; Qiagen, Hilden, Germany) in accordance with the manufacturer’s instructions. The* 16S-23S* rRNA ITS region in the extracted DNA samples was targeted for the diagnosis of *Bartonella* species [27]. During PCR analysis, a 489-bp fragment was amplified using the primer pairs 325s (5-CTTCAGATGATGATCCCAAGCCTTCTGGCG-3) and 1100as (5-GAACCGACGACCCCCTGCTTGCAAAGCA-3)(Eurofins Genomics Germany GmbH, Ebersberg, Germany)[27]. The amplification reaction mixture (30 µl) consisted of 5 µl template DNA, 1 µl of each primer (10 µM), 12.5 µl 2× PCR master mix (GeneMark, Taichung, Taiwan) and 10.5 µl nuclease-free water. PCR cycling program consisted of an initial denaturation of 2 min at 95 °C, followed by 35 cycles at 94 °C for 15 s, 66 °C for 15 s, and 72 °C for 15 s, with a final elongation at 72 °C for 1 min.

### Species identification

For species identification of *Bartonella* PCR-positive samples, sequences obtained by Sanger sequencing (Eurofins Genomics Germany GmbH) were aligned with MEGA 7.0 software and subject to BLAST analysis against the GenBank database. In addition, the obtained results also were confirmed by phylogenetic analysis performed by maximum likelihood method using the Kimura 2-parameter gamma distribution (K2 + G) model with 1000 bootstrap replications [28]. *Anaplasma phagocytophilum* was used as an outgroup. For sequences with identical nucleotides (100% identity), only one was used for phylogenetic analysis. The reference* 16S-23S* rRNA ITS sequences used in this study are given in Additional file 1: Table S1.

### Haplotype analysis

Haplotype analysis was performed using the DNASP program [29] using *Bartonella* isolates detected in this study and reference *B. henselae*, *B. clarridgeiae* and *B. koehlerae* strains isolated from cats in different countries. A haplotype network was generated in PopArt using the TCS network [30, 31]. For *Bartonella* species detected in the present study, the number of variable sites (VS), C + G content (GC%), number of haplotypes (h), haplotype diversity (Hd), nucleotide diversity (π), number of nucleotide differences (K) and standard deviation (SD) were calculated using the DNASP program. Sequences belonging to *B. henselae*, *B. clarridgeiae* and *B. koehlerae* from cats were retrieved in GenBank and used in the haplotype analysis. These included 24 *B. henselae* sequences from 10 countries (Spain, Malta, Brazil, Paraguay, Taiwan, Oklahoma, Guatemala, Korea, Australia and Malaysia), 40 *B. clarridgeiae* sequences from 13 countries (Spain, Malta, Portugal, Philippines, Brazil, Paraguay, China, USA, Taiwan, Indonesia, Japan, Greece and Iran), and five *B. koehlerae* sequences from two countries (Brazil and Malta).

### Statistical analysis

*Bartonella* positivity values detected in stray cats in Izmir were computed with the exact binomial confidence intervals of 95% (95% CI), and comparison of the proportions was performed by the Chi-square test using PASW Statistics version 18 software. Statistically significant differences were determined at *P* < 0.05.

## Results

### Molecular prevalence of *Bartonella* spp.

**Fig. 1:**
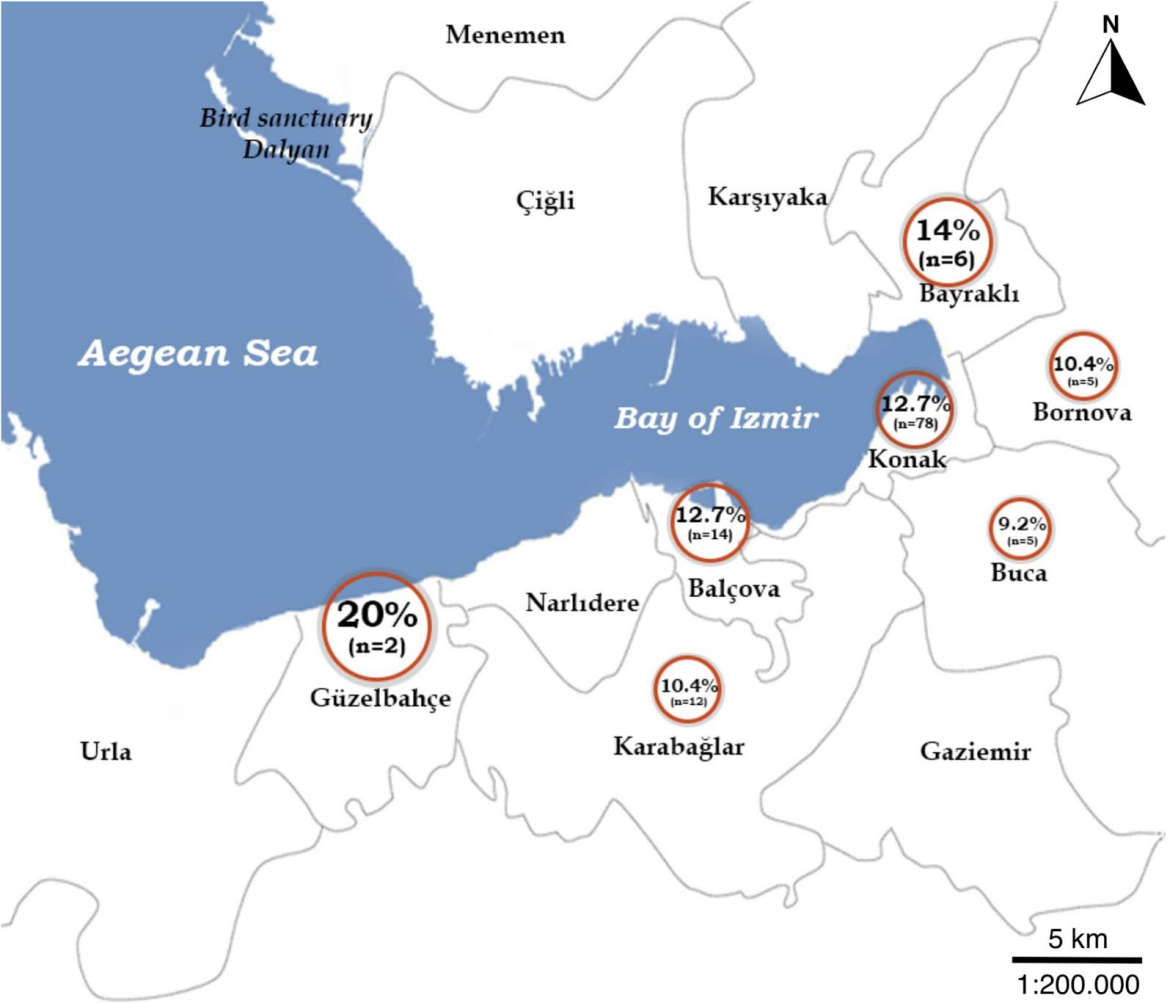
Map showing the prevalence of *Bartonella* PCR-positivity detected in sampled cats according to district of Izmir

DNA extracted from 1012 blood samples collected from stray cats was screened by PCR; of these 1012 DNA samples, 12.1% (122/1012; 95% CI 10.1–14.2%) were positive for *Bartonella* DNA. The highest prevalence was detected in samples collected from cats in Güzelbahçe (20%), followed by Bayraklı (14%) (Fig. 1). There was no statistically significant difference in detected *Bartonella* positivity values between the districts of Izmir sampled (Chi-square test, *χ*^2^ = 0.003, *df* = 1, *P* = *0.955*).

### Species identification

**Fig. 2:**
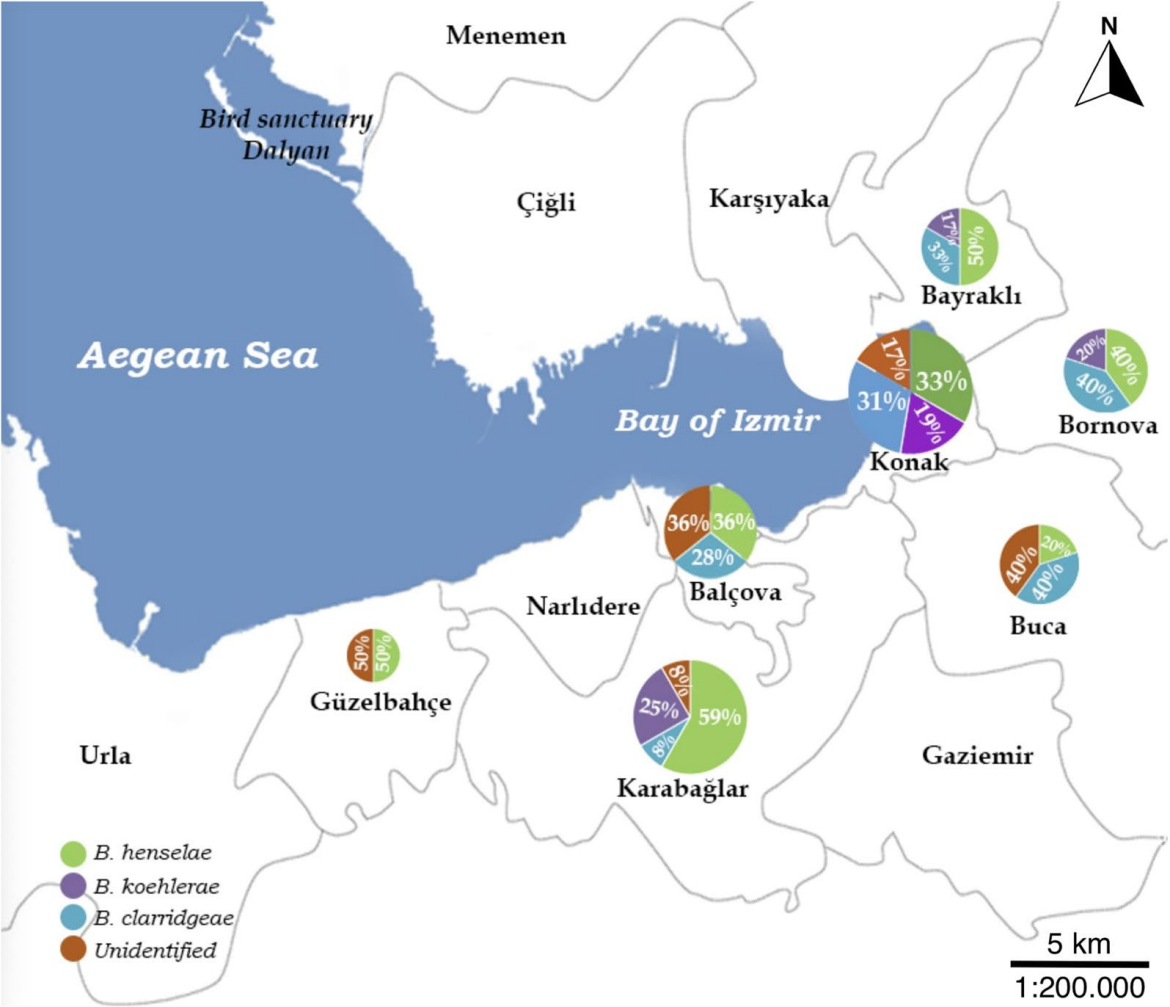
Map showing the distribution of *Bartonella* species detected in sampled cats according to district of Izmir

Among the *Bartonella*-positive samples, 100 (100/122; 81.96%) were successfully sequenced. BLAST and phylogenetic analyses revealed the presence of *B. henselae*, *B. clarridgeiae* and *B. koehlerae* in the positive samples. *Bartonella henselae* was the most common species detected in the stray cats (45%; 45/100) detected, followed by *B. clarridgeiae* (29%; 29/100) and then by *B. koehlerae* (26%; 26/100) (Fig. 2).

### Phylogenetic analysis and haplotype diversity

**Fig. 3:**
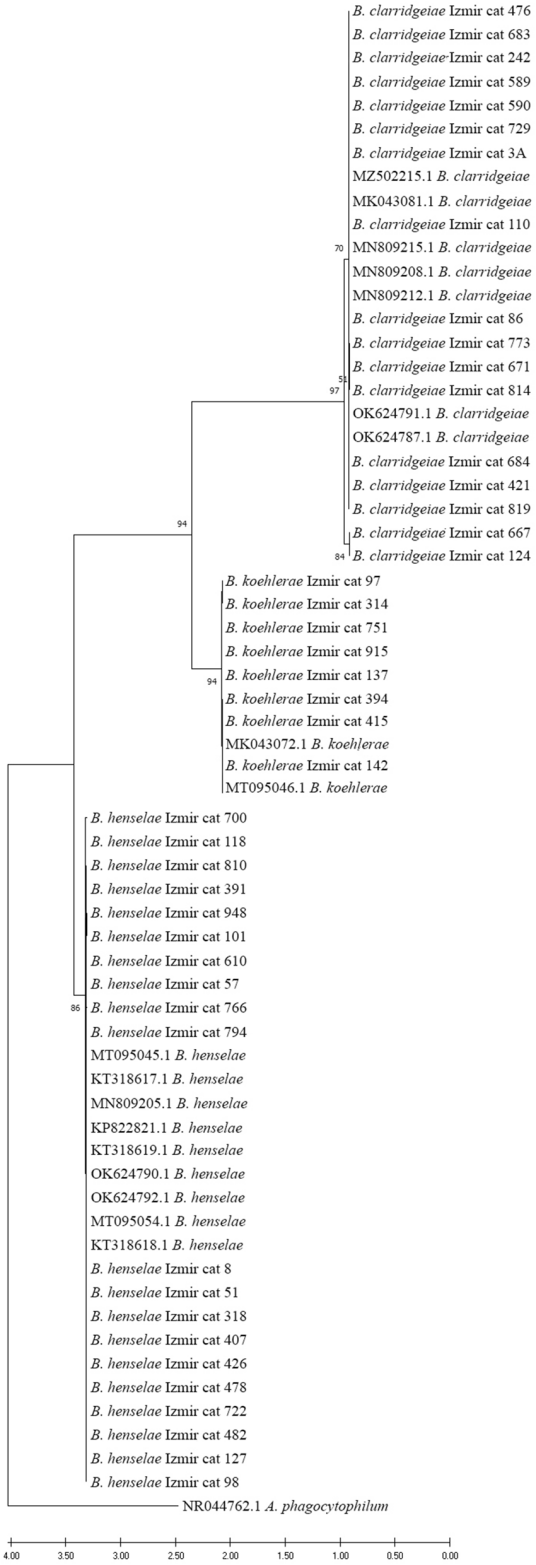
Phylogenetic tree shows the relationship of *Bartonella* species detected with reference *Bartonella* isolates. Phylogenetic analysis was performed by maximum likelihood method using the Kimura 2-parameter gamma distribution (K2 + G) model with 1000 bootstrap replications. *Anaplasma phagocytophilum* was used as an outgroup. Only bootstrap values > 50 are shown. Reference* 16S-23S* ribosomal RNA internal transcribed spacer sequences used in this study are given in Additional file 1: Table S1

All *Bartonella* species detected in this study clustered with reference sequences, forming well-defined groups separated by moderate and high bootstrap values (Fig. 3).

**Fig. 4:**
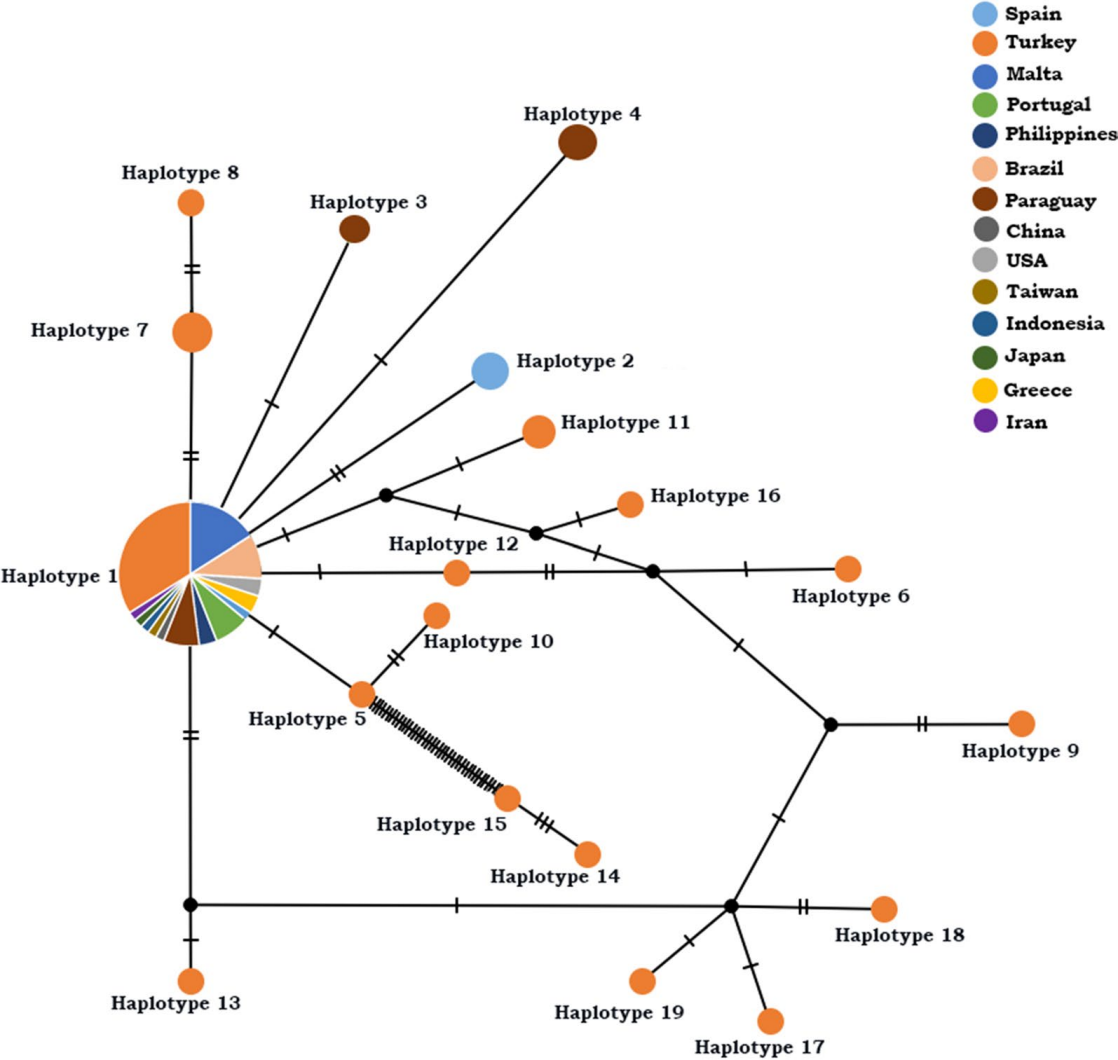
Haplotype analysis conducted for *Bartonella clarridgeiae* isolates. The haplotype network was generated in PopArt using the TCS network. According to this analysis, haplotype I containing 47 *B. clarridgeiae* isolated from 14 different countries, including Spain, Malta, Portugal, Philippines, Brazil, Paraguay, China, USA, Taiwan, Indonesia, Japan, Greece, Iran and Turkey, is the most prevalent haplotype. Haplotype I also represents the similar *Bartonella* isolates that are frequently detected in these countries. Each remaining haplotype represents unique *Bartonella* isolates to any country. Each color represents a country, as shown in Fig. 4

**Fig. 5:**
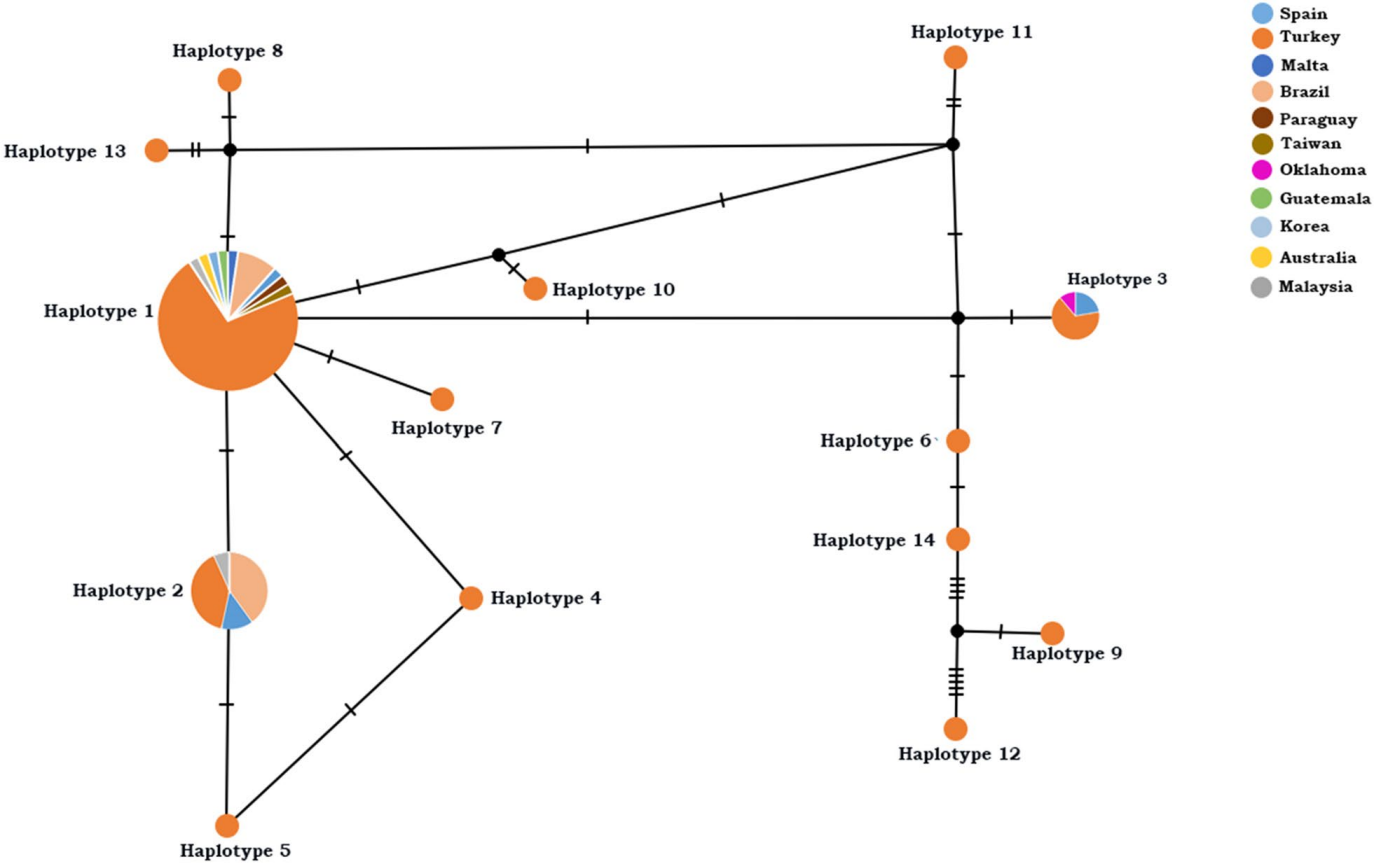
Haplotype analysis conducted for *Bartonella henselae* isolates. The haplotype network was generated in PopArt using the TCS network. According to this analysis, haplotype I containing 40 *B. henselae* isolated from 10 different countries, including Spain, Malta, Brazil, Paraguay, Taiwan, Guatemala, Korea, Australia, Malaysia and Turkey, is the most prevalent haplotype. Haplotype II and III containing more than one *Bartonella* isolate from different countries are among the prevalent haplotypes. Each remaining haplotype represents unique *Bartonella* isolates to any country. Each color represents a country, as shown in Fig. 5

**Fig. 6:**
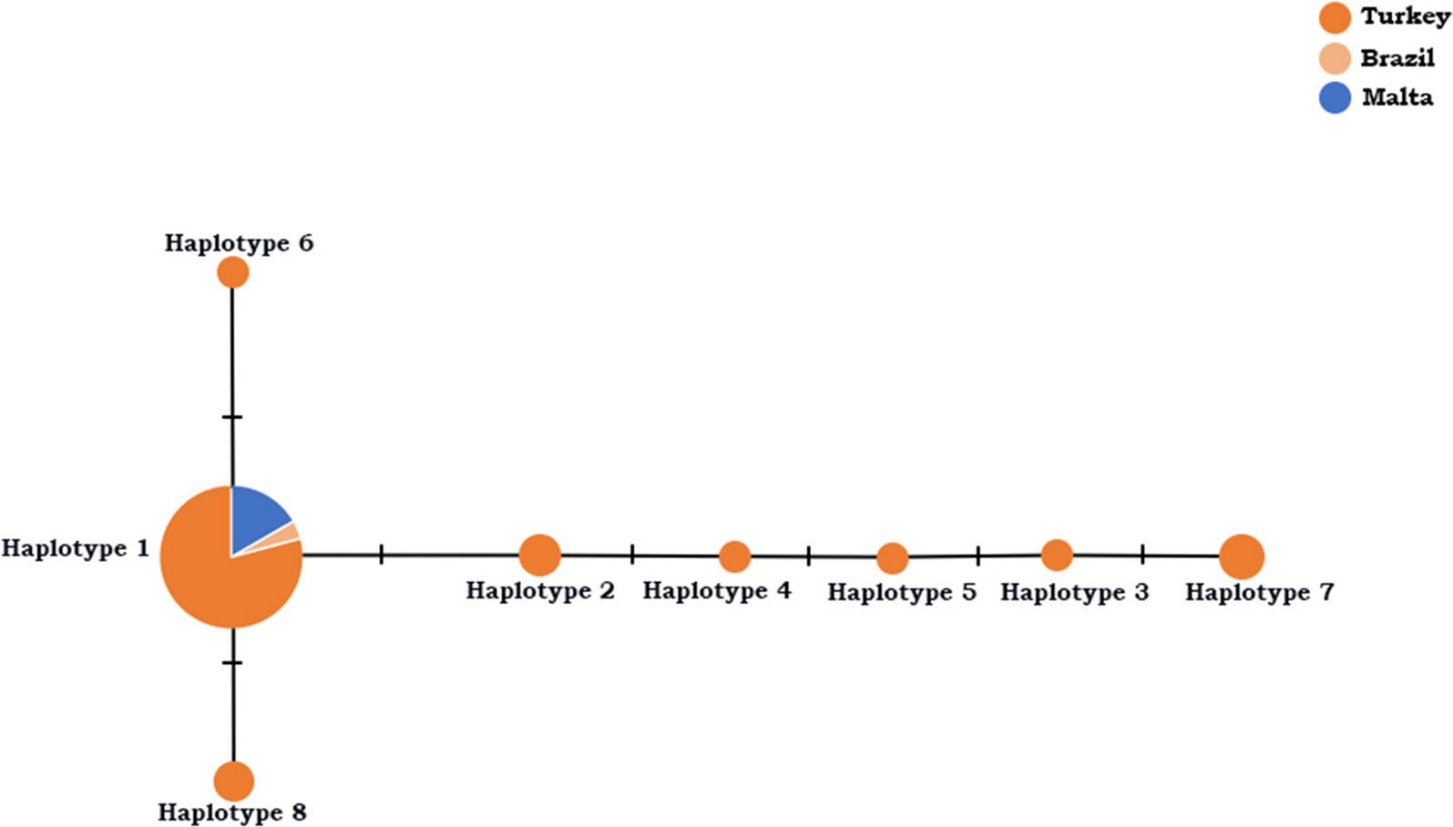
Haplotype analysis conducted for *Bartonella koehlerae* isolates. The haplotype network was generated in PopArt using the TCS network. According to this analysis, haplotype I containing 21 *B. koehlerae* isolated from three different countries, including Brazil, Malta and Turkey, is the most prevalent haplotype. This haplotype also represents the similar *Bartonella* isolates that are frequently detected in these countries. Each remaining haplotype represents unique *Bartonella* isolates to any country. Each color represents a country, as shown in Fig. 6

*Bartonella clarridgeiae* isolates (*n* = 69) belonged to 19 haplotypes (H-1 to H-19). Among these haplotypes, the most prevalent haplotype was H-1, which contained 47 *B. clarridgeiae* isolates from 14 countries, including Turkey (Fig. 4). The *B. clarridgeiae* sequences generated in this study belonged to different haplotypes only from Turkey (Fig. 4). Similarly, some *B. clarridgeiae* sequences from Paraguay and Spain also belonged to different haplotypes (Fig. 4). Haplotype analysis performed for *B. henselae* sequences (*n* = 59) belonged to 14 haplotypes (H-1 to H-14). Among these haplotypes, the most prevalent haplotype was H-1, which included 40 *B. henselae* sequences from 10 countries, including Turkey (Fig. 5). In addition to H-1, there were two haplotypes (H-2 and H-3) containing sequences from different countries (Fig. 5). Also, *B. henselae* detected in this study belonged to different haplotypes only from Turkey (Fig. 5). All *B. koehlerae* sequences (*n* = 31) belonged to eight haplotypes (H-1 to H-8). Among these haplotypes, the most prevalent haplotype was H-1, which contained 21 *B. koehlerae* sequences from three countries, including Turkey (Fig. 6). The *B. koehlerae* sequences detected in this study belonged to different haplotypes containing only isolates from Turkey (Fig. 6). The VS, GC%, h, Hd, π, K and SD for each *Bartonella* species detected in this study are presented in Table 1.

**Table 1:** Genetic diversity among *Bartonella* species detected in this study

*Bartonella* species	*N*	*VS*	*GC%*	*h*	*Hd*	*SD*	*π*	*K*
*B. clarridgeiae*	29	21	0.385	16	0.77586	0.00159	0.00812	3.18227
*B. henselae*	45	15	0.395	14	0.52929	0.00113	0.00287	1.12525
*B. koehlerae*	26	5	0.407	8	0.57231	0.00071	0.00248	0.97231

Only *Bartonella* samples detected in this study were used in the analysis

*VS* Number of variable sites,* GC%* C + G content,* h* number of haplotypes,* Hd* diversity of haplotypes,* n* nucleotide diversity,* K* number of nucleotide differences,* SD* standard deviation

## Discussion

In the present study we investigated the prevalence of *Bartonella* spp. in stray cats and identified the species of *Bartonella* present in the DNA collected from *Bartonella*-positive samples by sequencing. A haplotype analysis was also performed to reveal the genetic diversity of each *Bartonella* species detected. *Bartonella* DNA was detected in 12.1% of the samples collected from the stray cats. This prevalence is comparable with that reported in previous studies conducted in Turkey. In a study analyzing 256 samples from cats in Ankara, Turkey, *Bartonella* was detected in 9.4% of samples by blood culture [7] while the seroprevalence of *B. henselae* in the cats was 18.6%. Higher *Bartonella* prevalence values in cats also were reported in different studies using molecular or serological methods. Accordingly, the seroprevalence of *B. henselae* in cats was determined to be 41.3, 33.9, 27.5, 32.3, 17.9 and 12.5% in Bursa, Adana, Aydın, Burdur, Kayseri and Istanbul, respectively [8]. A study conducted in Tekirdağ reported a prevalence of 40.1% for *B. henselae* based on an analysis of samples collected from 167 client-owned symptomatic cats using PCR [32]*.* All of these results, obtained by blood culture, molecular or serological methods, indicate that *Bartonella* is prevalent in cats living in different locations of Turkey.

*Bartonella henselae*, *B. clarridgeiae* and *B. koehlerae* were the species detected in stray cats in this study. Among the *Bartonella-*positive samples, *B. henselae* was found to be the predominant species (prevalence: 45%) together with *B. clarridgeiae* and *B. koehlerae*. While less frequent than *B. henselae*, *B. clarridgeiae* is accepted as a causative agent for cat scratch disease [9] and *B. koehlerae* has been linked to endocarditis in humans [5].

The highest haplotype diversity was detected among *B. clarridgeiae* sequences*. Bartonella clarridgeiae* H-1 has been detected in Spain [33], Brazil [34], USA [35], Malta [36], Greece [37], Portugal [38], Philippines [39], Paraguay [40], Japan [41], Taiwan [39] and Indonesia [39]. Also, *B. henselae* H-1 has been reported in Spain [33], Malta [36], Brazil [42], Paraguay [40], Taiwan [33], Guatemala [43], Korea [44] and Australia [45]. *Bartonella henselae* H-2 was detected in Spain [33] and Brazil [42] whereas *B. henselae* H-3 has been reported in Spain [33]. Finally, *B. koehlerae* H-1 has been detected in Brazil [42] and Malta [36]. Within each *Bartonella* species, there were haplotypes that are apparently unique to Turkey in addition to haplotypes from different countries including Turkey (Figs. 4–6). Nonetheless, most of the *Bartonella* sequences obtained in this study belong to haplotypes that have also detected in cats in different countries.

Since previous studies carried out in Turkey reported anti-*B. henselae* antibodies in different human groups such as adult and pediatric patients [46], healthy blood donors [47], cattle breeders and veterinarians [48] and kidney transplant patients [49], stray cats could be an important source for transmission of *Bartonella* infection to humans in this country.

## Conclusion

In conclusion, we detected a 12.1% prevalence of *Bartonella* spp. infection in stray cats in Turkey, with *B. henselae*, *B. clarridgeiae* and *B. koehlerae* being the species detected.

## Supplementary Information

**Additional file 1: Table S1.** Reference *Bartonella* isolates used in phylogenetic tree and haplotype analysis.

## Data Availability

All sequences obtained from pathogens were deposited into GenBank (National Center for Biotechnology Information Search database) under GenBank accession numbers: ON673900-ON673928, ON673855-ON673899 and ON673929-ON673954.

## Abbreviations

CI: Confidence intervals
GC%: C + G content
h: Number of haplotypes
H: Haplotype
Hd: Haplotype diversity
ITS: Internal transcribed spacer
K: Average number of nucleotide difference
NCBI: National Center for Biotechnology Information
π: Nucleotide diversity index
rDNA: Ribosomal DNA
SD: Standard deviation
VS: Number of variable sites
