# Supplementary material for: Molecular prevalence and genetic diversity of Bartonella spp. in stray cats of İzmir, Turkey
Source: Parasit Vectors. 2022 Aug 29;15:305. doi: 10.1186/s13071-022-05431-3 (PMC9422166; doi:10.1186/s13071-022-05431-3)
Supplement: Supplementary file 1 — Additional file 1: Table S1. Reference Bartonella isolates used in phylogenetic tree and haplotype analysis. [file 13071_2022_5431_MOESM1_ESM.docx]

**Table S1.** Reference *Bartonella* isolates used in phylogenetic tree and haplotype analysis.

| GenBank accession numbers |
| --- |
| MK043076.1 |
| MK043079.1 |
| MK043077.1 |
| MK043080.1 |
| MK043081.1 |
| MK043074.1 |
| MK043073.1 |
| MK043071.1 |
| OK624793.1 |
| OK624791.1 |
| OK624787.1 |
| AB896695.1 |
| AB896698.1 |
| AB896696.1 |
| AB896697.1 |
| MZ323352.1 |
| KC331014.1 |
| KF977485.1 |
| OL449701.1 |
| KF977484.1 |
| KF977487.1 |
| KM067916.1 |
| MN809211.1 |
| MN809212.1 |
| MN809210.1 |
| MN809209.1 |
| MN809213.1 |
| MN809214.1 |
| MN809215.1 |
| MN809208.1 |
| EU589237.1 |
| DQ683194.1 |
| AF167989.1 |
| MZ323355.1 |
| MZ323353.1 |
| MZ323351.1 |
| AB674239.1 |
| MN170536.1 |
| MN170534.1 |
| MZ502215.1 |
| KT318618.1 |
| KT318619.1 |
| CP072899.1 |
| OK624788.1 |
| OK624792.1 |
| OK624790.1 |
| OK624784.1 |
| OK624789.1 |
| KP822821.1 |
| MT095054.1 |
| MT095051.1 |
| MT095045.1 |
| MT095052.1 |
| MT095053.1 |
| MT095050.1 |
| MT095049.1 |
| MT095048.1 |
| MT095047.1 |
| KC331013.1 |
| MZ323357.1 |
| MK043082.1 |
| JQ638927.1 |
| AJ441256.1 |
| MN809205.1 |
| MT095046.1 |
| MK043075.1 |
| MK043078.1 |
| MK043072.1 |
| MK043070.1 |
